# Inside a Metastatic Fracture: Molecular Bases and New Potential Therapeutic Targets

**DOI:** 10.1002/cam4.70901

**Published:** 2025-04-30

**Authors:** Alessandro Bruschi, Andrea Sambri, Michele Fiore, Elisa Bubbico, Cristina Scollo, Andrea Pace, Renato Zunarelli, Andrea Montanari, Alberta Cappelli, Lorenzo Di Prinzio, Massimiliano De Paolis

**Affiliations:** ^1^ Orthopedic and Traumatology Unit IRCCS Azienda Ospedaliero‐Universitaria di Bologna Bologna Italy; ^2^ Department of Biomedical and Neuromotor Sciences University of Bologna Bologna Italy; ^3^ Department of Medical and Surgical Sciences Alma Mater Studiorum University of Bologna Bologna Italy; ^4^ Department of Radiology IRCCS Azienda Ospedaliero‐Universitaria di Bologna Bologna Italy

**Keywords:** BMPs, bone metastasis, integrins, metastatic fracture, MMPs, NTx, PTHrP, RANK, skeletal related events

## Abstract

**Introduction:**

Bone metastases and pathological fractures significantly impact the prognosis and quality of life in cancer patients. However, clinical and radiological features alone have been shown to fail to predict skeletal related events of a bone metastasis (SREs).

**Aim:**

This study focuses on key molecular players including Matrix Metalloproteinases (MMPs), Integrins, Bone Morphogenetic Proteins (BMPs), Parathormone‐related Protein (PTHrP).

**Results:**

The RANK/RANKL/Osteoprotegerin (OPG) pathway, and N‐terminal peptide (NTx), involved in the metastatic process and bone integrity disruption. Elevated levels of these molecules have been pointed out as potential biomarkers for predicting SREs, but they have been poorly investigated. Moreover, batimastat, marimastat, tanomastat, andecaliximab, and HIV protease targeting MMPs; Volociximab/M200, cilengitide, abituzumab, and FAK inhibitors targeting integrins; LDN193189, DMH1, and ISLR modulators targeting BMPs; and PTH (7–33)‐CBD targeting PTHrP have shown promising results antagonizing these molecules, but no effect on preventing and managing metastatic fractures has been assessed yet.

**Conclusions:**

This paper underscores the importance of advanced molecular biology and transcriptomics in identifying novel therapeutic targets. The integration of these biomarkers with clinical and radiological assessments using artificial intelligence tools could revolutionize the diagnostics and treatment strategies for patients with bone metastases.

## 1 Introduction

Bone is the third most common metastatic site after lungs and liver [1]. Primary cancers most frequently causing bone metastases include breast cancer (bone metastasis prevalence of 25%–68%), lung cancer (5%–15%), kidney cancer (5%–18%), prostate cancer (8%–15%), and thyroid cancer (6%) [1, 2]. However, recent advancements in cancer treatment have led to improved survival rates, resulting in a higher incidence of bone metastatic disease. Consequently, bone metastases are now also reported in colon cancer (1.1% of 5479 in the study published by Baek et al. [3]), pancreatic cancer (2.2% as stated by Borad et al. [4]), melanoma (over 20% according to Wilson et al. [5]), urothelial cancer (32%–47% according to Shinagare et al. [6]) and hepatocellular carcinoma (around 2% as reported by Bhatia et al. [7]).

In bone metastatic process, cancer cells spread into the bone marrow space, creating secondary lesions by determining microenvironmental changes [8, 9]. Bone metastases can be classified as osteolytic, osteosclerotic, or mixed lesions [9]. Osteolytic metastases are more frequently seen due to metastatic involvement by breast, lung and kidney cancer. They are caused by tumor‐derived factors activating osteclasts, leading to increased bone resorption. Radiographically, these lesions usually appear as areas of bone lysis with cortical resorption. Histologically, tumor cells in the bone marrow cavity are surrounded by active osteoclasts. As cortical wall is destroyed by the osteolytic process, tumor cells can also infiltrate surrounding soft tissues [9, 10]. These areas with bone cortical weakening are prone to fractures even without trauma [10].

On the other side, osteosclerotic metastases result from cancer‐derived factors that stimulate osteoblasts to produce bone matrix. These are more commonly seen in prostate cancer; however, breast cancer can form osteosclerotic metastases as well. Radiographically, these lesions appear sclerotic, typically in vertebral bodies and pelvis [8, 9]. Histologically, tumor cells are surrounded by osteoblasts forming wide trabeculae of woven bone. However, the microstructure of this tumor‐associated woven bone is poorly organized, making the bone prone to pathological fracture as well [9, 10].

Bone resorption and formation are usually coupled processes. However, cancer disrupts this balance, leading to osteolytic or osteoblastic skeletal lesions. Nevertheless, many bone metastases are mixed lesions [9]. It is reported that a bone metastasis can transit from an osteoblastic to an osteolytic pattern through a continuous process, though this is only captured statically during radiographic or histological assessment [9, 11].

The destruction of bone by metastatic disease weakens its load‐bearing capabilities, initially causing microfractures that result in pain. This eventually leads to fractures, most commonly occurring in the ribs and vertebrae [11]. Fractures frequently occur in lytic lesions within weight‐bearing bones, where damage to both cortical and trabecular bone is structurally significant. Certain radiological features can predict an imminent fracture; these include large lesions, predominantly lytic lesions, and those that erode the cortex. Mirels proposed a scoring system based on the site, nature, size, and symptoms of a metastasis. Lesions scoring more than 7 typically require prophylactic surgical intervention, while those scoring 10 or higher have an estimated fracture risk of over 50% [12]. However, the predictive value of Mirels's score has been criticized by many studies for the high rates of unpredicted fractures, for the high inter‐/intra‐observer variation in the score and for the lack of reproducibility [2, 13, 14, 15, 16]. To date, no biomarker is used to predict skeletal related events (SREs, such as pathological fracture, spinal cord compression, bone pain, prophylactic surgical procedures, malignant hypercalcemia). Therefore, the aim of this study is to review the Literature to outline which biomarkers can integrate clinical and radiological assessment to predict metastatic bone involvement and metastatic fracture. This could be useful to prevent metastatic fracture and to define potential targets of medical treatment to reduce metastatic bone involvement.

## 2 Molecules Involved in Metastatic Bone Disease and Potential Therapeutic Antagonists

**FIGURE 1:**
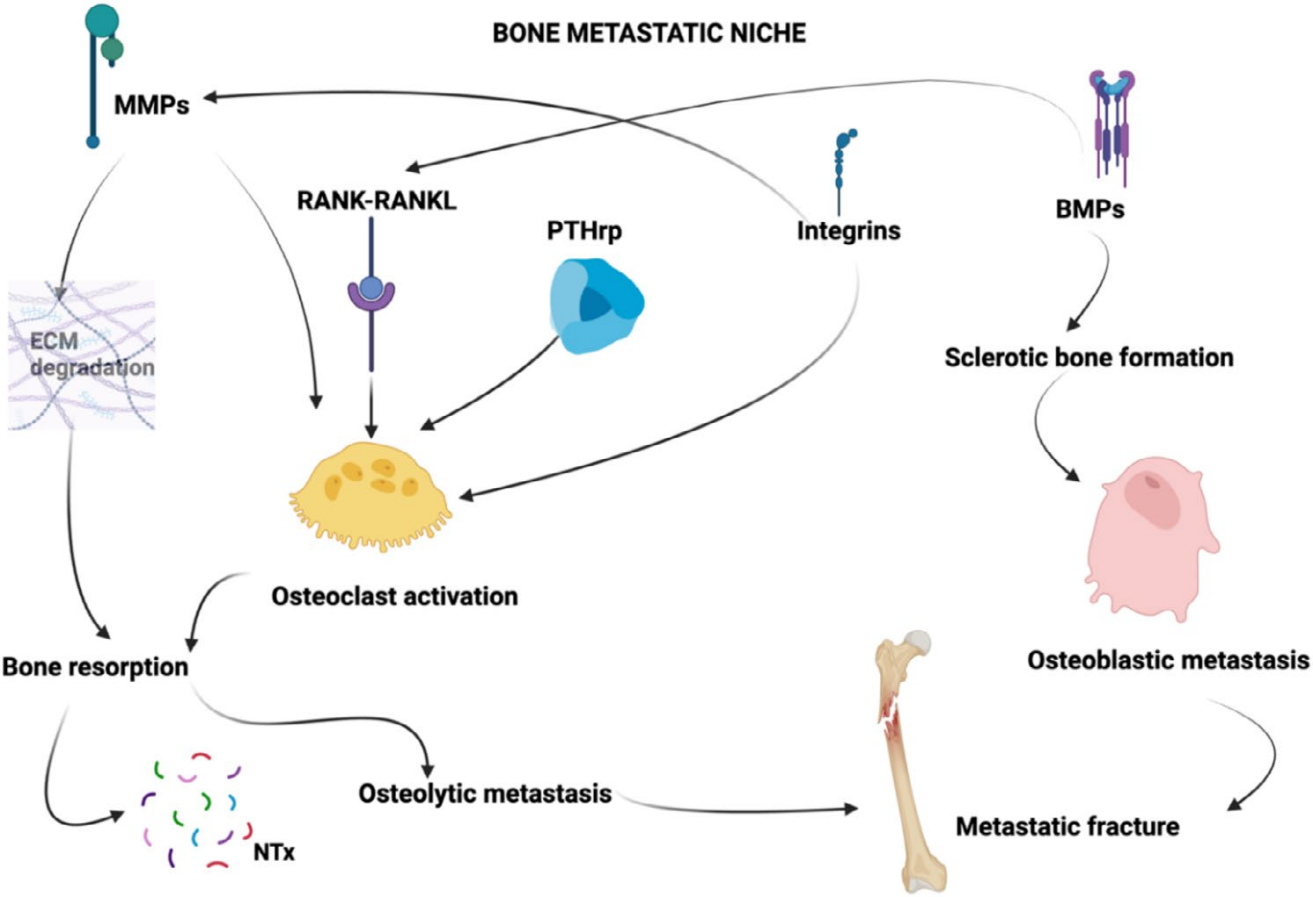
Schematic of molecular interplays inside the bone metastatic niche driving to a metastatic fracture. Matrix Metalloproteinases (MMPs) contribute to extracellular bone matrix (ECM) degradation and to osteoclast activation. The RANK‐RANKL pathway promotes osteoclast activation and it is enhanced by Bone Morphogenetic Proteins (BMPs). BMPs display a dual role, also activating osteoblasts in driving osteoblastic metastasis formation. On the other side, osteoclast activation is also promoted by Parathormone related peptide (PTH‐rP) and Integrins. This pathway drives bone resorption with N‐terminal peptide (NTx) formation and osteolytic metastasis. Both lytic and blastic metastasis are prone to fracture due to lower resistance of the pathological bone.

The Literature has identified many key molecules implicated in the process of bone metastasis, and they have emerged as promising candidates for predicting the risk of bone metastatic involvement and metastatic fractures [9, 17, 18, 19, 20, 21, 22, 23, 24, 25, 26, 27, 28] (Figure 1).

Some of these molecules have also been identified as potential targets for medical therapies [29, 30, 31, 32, 33, 34, 35, 36, 37, 38]. In this article we present some of the most reported ones; however, they may represent only a portion of a broader spectrum of biomarkers that need to be identified to increase the accuracy in the prediction of SREs and to serve as potential pharmacological targets for patients with bone metastases. These molecules exhibit a predominantly osteolytic pattern, like Metalloproteinases (MMPs), integrins, the RANK‐RANKL pathway, Parathormone related peptide (PTH‐rP), or a predominantly osteoblastic pattern like Bone Morphogenetic Proteins (BMPs), but their role can shift from osteolytic to osteoblastic and vice versa in particular cases depending on the specific microenvironment in which they are embedded [39, 40, 41, 42, 43]. Further research is therefore essential to expand our understanding of these biomarkers and to develop targeted therapies to effectively manage bone metastatic disease.

### 2.1 Matrix Metalloproteinases (MMPs)

MMPs are a group of proteases characterized by a structure that includes an amino‐terminal propeptide domain, a zinc‐containing catalytic site, a hemopexin domain providing substrate specificity, and a hinge region that connects the catalytic and hemopexin domains, giving the enzyme molecule flexibility [18]. Among them, MMP‐9 (also termed gelatinase B) plays a pivotal role in cancer growth and progression; therefore, it is considered a potential biomarker for the diagnosis and evolution of different types of cancers [18, 19, 44, 45, 46]. Actually, in breast cancer, non‐small cell lung cancer, ovarian cancer, pancreatic cancer, and osteosarcoma, the upregulation of MMP‐9 displays a crucial role in cancer progression and metastatization [40]. In fact, MMP‐9 plays a key role in the metastatic process by enhancing the tumor seeding and growth properties of the metastatic bone niche through extracellular matrix degradation, osteoclast activation, and by down‐regulating tumor‐infiltrating cytotoxic T lymphocytes and natural killer cells. In particular, the degradation of the extracellular matrix leads to the release of growth factors such as Transforming Growth Factor‐β (TGF‐β) and Insulin‐like Growth Factor (IGF), which contribute to osteoclast activation and bone resorption [47, 48, 49, 50, 51]. MMP‐9 also has a dual role in the activation of TGF‐β at the tumor‐bone interface in osteolytic lesions. First, through the degradation of the extracellular matrix, MMP‐9 indirectly releases TGF‐β. Second, MMP‐9 directly cleaves the Latent TGF‐β Binding Protein, thus releasing the active form of TGF‐β. This dual mechanism of MMP‐9, both by liberating TGF‐β through matrix degradation and by activating it via cleavage of its binding protein, significantly enhances TGF‐β signaling. Activated TGF‐β further stimulates tumor cells to produce PTHrP, which acts on osteoblasts to increase RANKL expression, leading to osteoclast activation and promoting bone resorption. This process contributes to the formation of osteolytic lesions observed in bone metastases [52].

Over the past 30 years, synthetic MMP inhibitors have been tested for cancer therapy [40]. These inhibitors, designed to chelate MMP catalytic zinc (like batimastat and marimastat) have shown promise in animal models but failed in later clinical trial phases due to poor bioavailability, pharmacokinetics, and musculoskeletal side effects [53]. Actually, first‐generation MMP inhibitors target all MMP family members simultaneously, disrupting essential physiological processes like wound healing [18, 53]. Consequently, using multiple drugs to inhibit all MMPs is not recommended [54]. Based on these clinical findings, structural analyses identified an exosite near the MMP active site that varies among MMPs and confers substrate binding specificity. New inhibitors, such as tanomastat, were designed to target this exosite and chelate the catalytic zinc in specific MMPs, including MMP‐9. However, these inhibitors have proven ineffective on overall survival and progression‐free survival of ovarian cancer patients; however, the drug was well tolerated and no information about the effect on skeletal involvement was reported [55]. In addition to these trials, efforts targeting MMP‐9 have shown that natural compounds like quercetin and curcumin can inhibit MMP‐9 expression or activity, enhancing chemotherapy efficacy and reducing side effects; however, effects on skeletal involvement were not analyzed [30, 31]. Humanized anti‐MMP‐9 monoclonal antibodies (andecaliximab) proved to be safe and moderately effective in gastric cancer patients when combined with traditional chemotherapy [56]. However, the cyclic peptide cilingitide (αv integrins antagonist) didn't inhibit glioblastoma progression; as well, the antibiotic doxycycline hasn't shown significant benefit in metastatic breast carcinoma patients, despite its effectiveness in animal models [57]. Furthermore, clinical trials have shown that combining HIV‐protease inhibitors (able to downregulate MMP‐9 function through the activation of the tissue inhibitors of MMPs, TIMPs) with standard chemo(radio)therapy promotes regression of advanced carcinomas in both HIV‐infected and uninfected individuals [58, 59]. However, HIV‐protease inhibitor monotherapy proved effective only against early‐stage tumors [60, 61, 62]. First‐generation HIV‐protease inhibitors (e.g., ritonavir, nelfinavir, and lopinavir) have multiple antitumor effects but can have metabolic side effects such as hyperglycemia and hyperlipidemia in patients [63]. Second‐generation inhibitors (e.g., darunavir and atazanavir) have less toxicity compared to first‐generation ones; however, their antitumor activities are not yet well defined [64]. In all the studies reported, the effect of the drug on the metastatic skeletal involvement was not studied as well as the dosage of MMPs, particularly MMP‐9, in predicting metastatic fractures. Our study underscores the crucial role of MMP‐9 in the pathogenetic cascade of bone metastases and emphasizes the need for further studies in this regard.

### 2.2 Integrins

Integrins play a role in cancer cell adhesion to the bone matrix and subsequent invasion, in activating osteoclasts and promoting MMPs activity [20, 21]. They also display a pivotal role in avoiding anoikis (programmed cell death due to the detachment from the surrounding ECM) by promoting cancer cell adhesion [7]. In 2021, Pantano et al. used in silico transcriptomic analyses of primary tumors and metastases to identify potential target genes involved in the spread of breast cancer to distant organs. Their study showed that integrin alpha5 (ITGA5) is highly expressed in bone metastases compared to non‐bone metastases. Additionally, multivariate analysis demonstrated that ITGA5 expression in primary breast tumors is an independent prognostic factor for bone relapse; their study identifies ITGA5 as a contributor to breast cancer metastasis to bone and suggests that volociximab/M200 might be repurposed for treating breast cancer patients with bone metastases who are ITGA5‐positive [65]. This outlines the importance of transcriptomic analysis to define gene expression in bone metastases and potential targets to be addressed by medical treatments [65, 66, 67]. Moreover, studies in mouse mammary tumor models and lung cancer show that integrin signaling promotes resistance to various therapies [68, 69, 70]. Integrins are attractive drug targets due to their accessible cell surface sites, but clinical trials have had limited success [71]. Notably, inhibitors targeting integrin‐ligand interactions, such as cilengitide and abituzumab, have failed to improve patient survival in trials in glioblastoma patients, but no data on skeletal involvement in other types of cancer is available [72]. New strategies may focus on tumor‐specific integrin profiles or downstream effectors: FAK, a protein activated by integrins, is linked to drug resistance and immunosuppression [32, 33, 73]. Current trials are investigating FAK inhibitors in combination therapies [7, 32, 74]. Integrins also modulate the tumor stroma, with LOX‐mediated matrix stiffening and bulky glycocalyx structures promoting integrin signaling and tumor survival [32]. Finally, no study has investigated the dosage of integrins in predicting metastatic fractures.

### 2.3 Bone Morphogenetic Proteins (BMPs)

BMPs are members of the transforming growth factor‐beta (TGF‐β) superfamily and play a role in bone formation and homeostasis [22]. BMP signaling demonstrates context‐dependent behavior, ranging from inhibition to tumor promotion. As the BMP pathway regulates the normal balance of stem cells, it often undergoes abnormal activation or repression depending on the microenvironment in which cancer stem cells (CSCs) reside [75]. CSCs, a small subset of tumor cells with stem‐like properties, play a critical role in driving tumor growth, managing therapy resistance, and contributing to recurrence [76]. Fully understanding the complex interactions between CSCs and their surrounding environment is crucial for developing effective treatments. Among the various signaling pathways that control CSCs' cellular behavior, BMP signaling stands out as a key regulator, guiding CSC self‐renewal, differentiation, and the complex interactions within the tumor microenvironment [77]. Additionally, BMP signaling's role in cancer extends beyond CSCs, intricately controlling cellular migration, invasion, and metastasis [34]. BMP signaling exhibits context‐dependent pleiotropic effects in different cancers. For instance, in lung, colorectal, prostate, and breast cancers, BMP signaling can drive tumorigenesis and metastasis, while in others, such as Glioblastoma Multiforme (GBM), BMP signaling suppresses tumor stemness by promoting the differentiation of Glioblastoma stem cells (GSCs) into an astroglial fate, thus inhibiting tumor growth progression [78]. A study has demonstrated that the BMP antagonist, COCO, plays a crucial role in modulating the reawakening of dormant metastatic breast tumors linked to CSCs in the lung, while BMP signaling itself exerts suppressive effects [79]. On the other hand, regarding the promotion of organ‐specific tumor metastasis, the role of BMPs in bone metastasis is particularly well‐documented in prostate cancer. In vitro studies have highlighted the combined effect of BMP4 and SHH in supporting the survival of prostate cancer cells and promoting the differentiation of bone stromal cells, which may lead to the osteoblastic metastasis typical of prostate cancer [80]. Furthermore, in vivo research has emphasized BMP4's role in bone formation within a xenograft model of prostate cancer bone metastasis. Importantly, blocking BMP receptors with LDN193189 has been shown to hinder osteoblast differentiation and reduce tumor growth [43].

Recent studies have highlighted the potential of inhibiting systemic BMP signaling as a strategy to stop tumor progression and metastasis, targeting both the tumor and its surrounding microenvironment. A significant example is the use of dorsomorphin (DMH1), a BMP antagonist, which acts by blocking the activation of BMP type I receptors, preventing their activation. These receptors are essential for BMP signal transduction in cells by promoting phosphorylation of SMAD proteins (particularly SMAD1, SMAD5, and SMAD8) [35]. In this way, DMH1 prevents transcription of genes that regulate cell differentiation, growth, and bone formation. DMH1 has demonstrated promising results: DMH1 treatment has been effective in reducing lung metastases in breast cancer and in human xenograft lung cancer models [34, 35, 81]. Additionally, in vivo studies showed a decrease in tumor proliferation and an increase in apoptosis, underscoring the therapeutic potential of targeting BMP signaling [81]. In this 2021 study, the Authors tested a modulator of the effect of BMPs on the metastatic niche (ISLR) obtaining a prolonged survival of mice in which the drug was tested compared to the control group [82]. ISLR was on the other side evaluated as a potential target for osteoporosis in mice in this 2024 study, where ISLR knockout mice showed enhanced bone formation [83]. Actually, ISLR knockout led to increased osteogenic differentiation and mineral accumulation; ISLR acts as a negative regulator of osteogenic differentiation via the BMP4‐Smad‐ColIα1/Ocn pathway; ISLR interacts with BMP4 to influence osteogenic differentiation, facilitating the proteasomal breakdown of BMP4; targeted deletion of ISLR in osteoblasts resulted in higher bone density in Ocn‐cre/flox mice, whereas mice with overexpressed ISLR exhibited reduced bone mass; therefore, ISLR was identified as a new regulatory factor in osteogenic differentiation and bone growth [83]. However, the effect on skeletal metastases was not specifically assessed in any study as well as the potential use of BMPs in the prediction of metastatic fractures.

### 2.4 Parathormone Related Protein (PTHrP)

PTHrP acts by binding to the same receptor as parathyroid hormone (PTH), namely the PTH/PTHrP receptor (PTH1R), which is abundantly expressed on osteoblasts and osteoclasts [23, 24]. In the bone microenvironment, PTHrP promotes osteoclastogenesis indirectly by stimulating osteoblasts to produce receptor activator of nuclear factor kappa‐Β ligand (RANKL), a crucial factor in osteoclast differentiation and activation [23, 24]. This leads to increased bone resorption, creating a favorable niche for tumor cells to colonize and expand within the bone; in this study, it has been reported that in breast cancer, 90% of metastatic cells express PTHrP, while only 50% of cells of the primary tumor express it, underscoring the critical role of PTHrP in the skeletal involvement of breast cancer. The role of PTHrP in bone metastases is not merely confined to bone resorption [84]. Emerging evidence suggests that PTHrP also contributes to the osteoblastic response in certain cancers, such as prostate cancer, where bone metastases are often osteoblastic rather than purely osteolytic [36, 37]. In this context, PTHrP may promote an aberrant bone formation process, which, although seemingly paradoxical, still contributes to skeletal morbidity by leading to structurally abnormal and brittle bone [37]. Clinically, the presence of high levels of PTHrP in patients with bone metastases has been pointed out as a biomarker for increased fracture risk [36, 85]. Moreover, the local production of PTHrP within bone metastases suggests that targeting this pathway could be a viable therapeutic strategy [9, 38]. For instance, inhibitors of PTHrP signaling, or agents that block RANKL, such as denosumab, have shown promise in reducing skeletal‐related events in cancer patients by decreasing bone turnover and preserving bone integrity [38]. The understanding of PTHrP's role in bone metastases and pathological fractures opens several avenues for therapeutic intervention. Current treatments targeting the bone microenvironment, including bisphosphonates and denosumab, indirectly affect PTHrP‐mediated pathways [29, 38, 86, 87]. However, more specific inhibitors of PTHrP or its downstream signaling molecules could potentially offer more targeted and effective treatments for preventing skeletal complications in cancer patients [88, 89]. Furthermore, future research should focus on elucidating the precise mechanisms by which PTHrP contributes to both osteolytic and osteoblastic processes in bone metastases. A deeper understanding of these pathways could lead to the development of novel therapies: a monoclonal antibody targeting PTHrP represents a promising therapeutic strategy in combating breast cancer, particularly in aggressive forms like triple‐negative breast cancer (TNBC) in which PTHrP has shown a role in brain metastases development, but no effect on skeletal involvement was mentioned [88]. On the other side, in this 2021 study, PTH (7–33)‐CBD, a novel PTHrP antagonist, has shown to reduce bone metastasis and prevent osteolytic metastases in metastatic breast cancer mice models [90].

In conclusion, PTHrP plays a multifaceted role in the development of bone metastases and pathological fractures in carcinoma patients. Its contribution to both bone resorption and abnormal bone formation underscores its strong potential as a molecule to dose to predict metastatic fractures and as a therapeutic target. Continued research in this area is essential for improving the management of skeletal complications in cancer patients, ultimately enhancing their quality of life and clinical outcomes.

### 2.5 RANK/RANKL/Osteoprotegerin (OPG)

Receptor Activator of Nuclear Factor Kappa‐B (RANK) and its molecular signaling cascade play a critical role in the development of bone metastases and the resulting pathological fractures observed in advanced cancers [25, 26, 27]. RANK is a receptor expressed on the surface of osteoclast precursors and mature osteoclasts, which are the cells responsible for bone resorption. Its primary ligand, RANKL, is produced by osteoblasts, stromal cells, and even cancer cells in the bone microenvironment [25, 91]. The binding of RANKL to RANK is a critical step in osteoclast differentiation, activation, and survival [91]. This interaction promotes the maturation of osteoclasts from their precursors and stimulates bone resorption, a process essential for normal bone remodeling but detrimental when dysregulated in the context of bone metastasis [25, 91].

The RANK/RANKL/OPG (osteoprotegerin) axis is tightly regulated under normal physiological conditions, where OPG acts as a decoy receptor for RANKL, preventing its interaction with RANK and thereby inhibiting osteoclastogenesis [91]. However, in cancer, the balance of this axis is often disrupted [26]. Tumor cells can produce RANKL or induce stromal cells to increase RANKL production while simultaneously decreasing OPG levels, leading to enhanced osteoclast activity and bone resorption [91, 92]. This creates an environment conducive to bone metastasis, as the resorption process releases growth factors from the bone matrix that further promote tumor cell proliferation and survival.

The RANK/RANKL/OPG pathway is central to the vicious cycle of bone metastasis and metastatic fractures: as tumor cells invade the bone, they disrupt normal bone homeostasis by increasing RANKL expression, which in turn enhances osteoclast‐mediated bone destruction; this not only facilitates the growth of the metastatic tumor within the bone but also leads to the deterioration of bone integrity, making it prone to fractures even under normal stress [93].

Therapeutically, targeting the RANK/RANKL pathway has shown significant promise in treating bone metastases and preventing SREs. Denosumab, a monoclonal antibody that binds to RANKL, effectively inhibits its interaction with RANK, thus preventing osteoclast activation [29, 38, 94]. Clinical trials have demonstrated that denosumab reduces the incidence of SREs, including pathological fractures, in patients with bone metastases from solid tumors [95, 96]. This highlights the critical role of the RANK/RANKL pathway in the pathophysiology of bone metastasis and underscores the potential of targeting this pathway to improve outcomes in patients with metastatic bone disease [29, 38, 94]. As well as denosumab, bisphosphonates represent a cornerstone in the management of metastatic bone involvement, showing reduced SREs [86, 87, 97, 98]. Despite not directly targeting RANKL, they interfere with the RANK/RANKL/OPG chain by inhibiting osteoclast function by two main mechanisms: (1) Non‐Nitrogen‐Containing Bisphosphonates (N‐NBPs): these compounds, such as etidronate and clodronate, are metabolized by osteoclasts into non‐hydrolyzable ATP analogs, which accumulate within the cell and lead to apoptosis. This disrupts the energy metabolism of osteoclasts, effectively reducing bone resorption; (2) Nitrogen‐Containing Bisphosphonates (N‐BPs): drugs like alendronate, zoledronate, and risedronate belong to this group. They inhibit the mevalonate pathway, a crucial metabolic pathway in osteoclasts, which leads to the disruption of protein prenylation—a process necessary for the function of small GTPase signaling proteins. This disruption causes osteoclast apoptosis, thereby inhibiting bone resorption more effectively than N‐NBPs [97, 98]. Interestingly, prostate cancer bone metastases also have a lytic component underlying their osteoblastic phenotype, with RANK/RANKL/OPG playing an important role [86]; actually, denosumab and bisphosphonates have been positively used also to reduce SREs in prostate cancer [86, 99].

Therefore, the RANK signaling cascade is crucial to the process of bone metastasis and the development of pathological fractures. By promoting osteoclast‐mediated bone resorption, the RANK/RANKL interaction not only facilitates tumor growth within the bone but also leads to the structural weakening of bones, resulting in fractures. Targeting this pathway is the gold standard approach to manage bone metastases and reduce the incidence of SREs in cancer patients. However, both denosumab and bisphosphonates are burdened by side effects like jaw osteonecrosis, hypocalcemia, urinary tract infections, dermatitis, hypercholesterolemia, and atypical fractures [87, 100]; moreover, RANK, as shown in this article, is not the only pivotal factor in the bone metastatic process; thus, molecular biology and transcriptomic analysis may be crucial in defining new molecular targets other than the RANK/RANKL/OPG pathway, optimizing bone metastases treatment [65, 66].

### 2.6 N‐Terminal Peptide (NTx)

NTx, a byproduct of type I collagen degradation, serves as a biomarker for osteoclastic activity and bone resorption. Its serum and/or urine dosage can play a significant role in the context of bone metastases and pathological fractures, providing critical insights into the processes of bone resorption and turnover [101].

In patients with bone metastases, elevated levels of NTx are indicative of increased osteoclastic activity [102]. This heightened resorption activity can weaken the bone structure, predisposing patients to pathological fractures [17, 28]. Monitoring NTx levels can thus provide valuable prognostic and diagnostic information in the management of patients with cancers that have a high propensity to metastasize to bone [17, 101, 103, 104].

The role of NTx extends beyond mere indication of bone turnover. It also offers insights into the efficacy of treatments aimed at reducing skeletal‐related events (SREs) in cancer patients. Therapies that target bone resorption, such as bisphosphonates and denosumab, aim to mitigate the osteoclastic activity that leads to high NTx levels [105, 106]. By monitoring changes in NTx levels, clinicians can assess the therapeutic response, adjusting treatment plans to optimize patient outcomes and potentially prevent the occurrence of pathological fractures [42, 107, 108].

Furthermore, understanding the dynamics of NTx in the bone microenvironment helps in deciphering the mechanisms through which bone metastases facilitate tumor growth and progression. The interaction between cancer cells and bone cells can stimulate the production of factors that increase bone resorption, thereby elevating NTx levels [103, 104]. This interaction not only aids in the diagnosis and management of bone metastases but also enhances our understanding of the metastatic process itself, offering potential avenues for targeted therapeutic interventions [42, 107, 109].

In summary, NTx might serve as a crucial biomarker in the management of bone metastases and in the prevention of metastatic fractures. Its role in reflecting osteoclastic activity provides essential insights into bone health in patients with advanced cancers, guiding therapeutic strategies and contributing to a more comprehensive understanding of bone metastasis biology (Table 1).

**TABLE 1:** Summary of the role of the molecules analyzed in the text: role in bone metastasis, associated therapeutic agents, clinical phase for approval of the therapeutic agents, limitation of the therapeutic agents.

Molecule	Role in bone metastasis	Associated drugs	Clinical trial phase	Main limitations
MMP‐9	Bone matrix degradation, osteoclast activation, bone resorption	MMP inhibitors (marimastat, batimastat); humanized anti‐MMP‐9 monoclonal antibodies; HIV‐protease inhibitors	Preclinical and clinical studies [53, 55, 56]	Toxicity, poor specificity, lack of trials on bone metastasis
Integrins	Tumor cell adhesion to bone matrix, osteoclast activation, promoting MMPs activity	Integrin inhibitors (cilengitide, abituzumab); FAK inhibitors	Clinical studies [33, 72, 73]	Limited efficacy, difficulty in specific targeting, lack of trials on bone metastasis
BMPs	Promoting the differentiation of bone stromal cells, sclerotic bone formation	BMP antagonist (DMH1); modulator of the effect of BMPs on metastatic niche (ISLR)	Preclinical studies [35, 39]	Dual roles of BMPs (tumor suppression vs. pro‐ metastatic signaling), lack of trials on bone metastasis
PTHrP	Stimulates osteoblasts to produce RANKL, promoting osteoclastogenesis, bone resorption	Denosumab (anti‐RANKL); PTHrP antagonist (PTH (7–33)‐CBD)	Denosumab: approved; [29, 38, 94, 95] preclinical study [90]	Side effects, lack of trials on bone metastasis
RANK/RANKL	Regulation of osteoclastogenesis, bone resorption	Denosumab (anti‐RANKL), bisphosphonates	Approved for clinical use [87]	Side effects: jaw osteonecrosis, hypocalcemia, urinary tract infections, dermatitis, hypercholesterolemia, and atypical fractures
NTx	Biomarker for osteoclastic activity and bone resorption. Monitors the efficacy of bisphosphonate or denosumab treatment	—	—	Limited to diagnostic/prognostic role

## 3 Conclusion

Nowadays, clinical and radiological features alone fail to predict skeletal related events of a bone metastasis. This study underscores the pivotal role of MMPs, integrins, BMPs, PTHrP, RANK, and NTx in bone metastatic involvement and metastatic fracture. Their dosage can be useful to integrate clinical and radiological features to predict skeletal related events. To date, batimastat, marimastat, tanomastat, andecaliximab, and HIV protease for MMPs, Volociximab/M200, cilengitide, abituzumab, and FAK inhibitors for integrins, LDN193189, DMH1, and ISLR modulators for BMPs, PTH (7–33)‐CBD for PTHrP have shown promising results in targeting these molecules, but no effect on preventing and managing metastatic fractures has been assessed yet. Therefore, studies outlining their effects on skeletal related events can be a turning point in the treatment of bone metastases, aside from denosumab and bisphosphonates that only target the RANK/RANKL/OPG cascade and have several side effects. Finally, molecular biology and transcriptomics are crucial in identifying new genes and potential therapeutic targets involved in bone metastases and metastatic fractures. Including these data in artificial intelligence tools considering clinical, radiological, and biomarkers features can offer more precise diagnostics and therapeutic possibilities to patients suffering from bone metastases.

## Author Contributions

**Alessandro Bruschi:** conceptualization (equal), data curation (equal), investigation (equal), methodology (equal), supervision (equal), visualization (equal), writing – original draft (equal), writing – review and editing (equal). **Andrea Sambri:** conceptualization (equal), data curation (equal), project administration (equal), validation (equal), writing – review and editing (equal). **Michele Fiore:** conceptualization (equal), formal analysis (equal), funding acquisition (equal), methodology (equal), resources (equal), validation (equal). **Elisa Bubbico:** conceptualization (equal), formal analysis (equal), methodology (equal), resources (equal), validation (equal). **Cristina Scollo:** investigation (equal), methodology (equal), project administration (equal), resources (equal), software (equal). **Andrea Pace:** data curation (equal), formal analysis (equal), writing – review and editing (equal). **Renato Zunarelli:** data curation (equal), writing – review and editing (equal). **Andrea Montanari:** data curation (equal), software (equal). **Alberta Cappelli:** conceptualization (equal), investigation (equal), validation (equal), visualization (equal). **Lorenzo Di Prinzio:** data curation (equal), visualization (equal). **Massimiliano De Paolis:** conceptualization (equal), investigation (equal), project administration (equal), supervision (equal), validation (equal), writing – review and editing (equal).

## Conflicts of Interest

The authors declare no conflicts of interest.

## Data Availability

This study did not generate or analyze any datasets.
